# Supplementary material for: Multi‐dataset identification of innovative feature genes and molecular mechanisms in keratoconus
Source: J Cell Mol Med. 2024 Sep 19;28(18):e70079. doi: 10.1111/jcmm.70079 (PMC11412914; doi:10.1111/jcmm.70079)
Supplement: Supplementary file 2 — Material S2. [file JCMM-28-e70079-s002.zip › 7.GSEA analysis/3.DBNDD1/KEGG_GLYCOLYSIS_GLUCONEOGENESIS.html]

Details for gene set KEGG\_GLYCOLYSIS\_GLUCONEOGENESIS[GSEA]

|  || Dataset | DBNDD1.group.cls#h\_versus\_l |
| Phenotype | group.cls#h\_versus\_l |
| Upregulated in class | l |
| GeneSet | KEGG\_GLYCOLYSIS\_GLUCONEOGENESIS |
| Enrichment Score (ES) | -0.40913832 |
| Normalized Enrichment Score (NES) | -1.4716716 |
| Nominal p-value | 0.04904051 |
| FDR q-value | 0.5773516 |
| FWER p-Value | 0.832 |
Table: GSEA Results Summary

  

Fig 1: Enrichment plot: KEGG\_GLYCOLYSIS\_GLUCONEOGENESIS      
 Profile of the Running ES Score & Positions of GeneSet Members on the Rank Ordered List

  

| SYMBOL | TITLE | RANK IN GENE LIST | RANK METRIC SCORE | RUNNING ES | CORE ENRICHMENT || 1 | ADH6 | na | 3734 | 0.222 | -0.0694 | No |
| 2 | ALDOC | na | 5665 | 0.159 | -0.0977 | No |
| 3 | ADH1B | na | 5723 | 0.158 | -0.0721 | No |
| 4 | LDHAL6A | na | 6564 | 0.135 | -0.0731 | No |
| 5 | AKR1A1 | na | 7373 | 0.116 | -0.0763 | No |
| 6 | ALDH3B1 | na | 8402 | 0.096 | -0.0894 | No |
| 7 | FBP1 | na | 9407 | 0.080 | -0.1047 | No |
| 8 | PCK1 | na | 9869 | 0.073 | -0.1054 | No |
| 9 | ACSS1 | na | 9886 | 0.073 | -0.0933 | No |
| 10 | DLD | na | 11443 | 0.053 | -0.1291 | No |
| 11 | LDHC | na | 12007 | 0.047 | -0.1373 | No |
| 12 | ALDH3A2 | na | 12835 | 0.040 | -0.1543 | No |
| 13 | PGM2 | na | 13183 | 0.037 | -0.1580 | No |
| 14 | ENO1 | na | 14910 | 0.023 | -0.2037 | No |
| 15 | PFKP | na | 15214 | 0.021 | -0.2088 | No |
| 16 | PDHA2 | na | 15714 | 0.018 | -0.2202 | No |
| 17 | PDHB | na | 16264 | 0.014 | -0.2336 | No |
| 18 | ACSS2 | na | 16863 | 0.010 | -0.2491 | No |
| 19 | PFKL | na | 17306 | 0.007 | -0.2605 | No |
| 20 | BPGM | na | 17592 | 0.006 | -0.2678 | No |
| 21 | ADH7 | na | 17985 | 0.003 | -0.2786 | No |
| 22 | ALDH7A1 | na | 18637 | -0.002 | -0.2971 | No |
| 23 | PGAM2 | na | 19251 | -0.006 | -0.3136 | No |
| 24 | DLAT | na | 19269 | -0.007 | -0.3130 | No |
| 25 | TPI1 | na | 19904 | -0.012 | -0.3292 | No |
| 26 | GAPDH | na | 20072 | -0.014 | -0.3316 | No |
| 27 | ADH5 | na | 20394 | -0.016 | -0.3381 | No |
| 28 | PGAM4 | na | 20530 | -0.018 | -0.3389 | No |
| 29 | PGAM1 | na | 20968 | -0.022 | -0.3478 | No |
| 30 | HK1 | na | 21104 | -0.023 | -0.3477 | No |
| 31 | PGK1 | na | 21105 | -0.023 | -0.3438 | No |
| 32 | PKM | na | 21378 | -0.026 | -0.3472 | No |
| 33 | ALDOA | na | 21556 | -0.027 | -0.3476 | No |
| 34 | PFKM | na | 22338 | -0.035 | -0.3640 | No |
| 35 | ENO2 | na | 22371 | -0.036 | -0.3587 | No |
| 36 | ALDH1B1 | na | 22410 | -0.036 | -0.3536 | No |
| 37 | LDHB | na | 22462 | -0.037 | -0.3487 | No |
| 38 | ALDH9A1 | na | 22786 | -0.041 | -0.3510 | No |
| 39 | ALDH3A1 | na | 23496 | -0.049 | -0.3630 | No |
| 40 | PGM1 | na | 23581 | -0.051 | -0.3567 | No |
| 41 | PDHA1 | na | 24150 | -0.058 | -0.3630 | No |
| 42 | LDHA | na | 24317 | -0.061 | -0.3574 | No |
| 43 | ENO3 | na | 24958 | -0.070 | -0.3638 | No |
| 44 | GPI | na | 25733 | -0.082 | -0.3719 | No |
| 45 | G6PC2 | na | 26032 | -0.088 | -0.3653 | No |
| 46 | HK3 | na | 26158 | -0.091 | -0.3533 | No |
| 47 | ALDH3B2 | na | 28095 | -0.133 | -0.3862 | Yes |
| 48 | LDHAL6B | na | 28137 | -0.134 | -0.3643 | Yes |
| 49 | GCK | na | 28250 | -0.136 | -0.3440 | Yes |
| 50 | ALDOB | na | 28498 | -0.143 | -0.3265 | Yes |
| 51 | GALM | na | 29635 | -0.177 | -0.3288 | Yes |
| 52 | HK2 | na | 29828 | -0.183 | -0.3029 | Yes |
| 53 | ADH1A | na | 29956 | -0.187 | -0.2743 | Yes |
| 54 | PKLR | na | 30152 | -0.193 | -0.2467 | Yes |
| 55 | FBP2 | na | 31299 | -0.233 | -0.2397 | Yes |
| 56 | ALDH1A3 | na | 31867 | -0.257 | -0.2118 | Yes |
| 57 | PCK2 | na | 32336 | -0.284 | -0.1764 | Yes |
| 58 | ADH4 | na | 32701 | -0.306 | -0.1341 | Yes |
| 59 | ALDH2 | na | 33235 | -0.347 | -0.0897 | Yes |
| 60 | ADH1C | na | 33609 | -0.380 | -0.0350 | Yes |
| 61 | PGK2 | na | 33701 | -0.391 | 0.0296 | Yes |
Table: GSEA details [plain text format]

  

Fig 2: KEGG\_GLYCOLYSIS\_GLUCONEOGENESIS      
 Blue-Pink O' Gram in the Space of the Analyzed GeneSet

  

Fig 3: KEGG\_GLYCOLYSIS\_GLUCONEOGENESIS: Random ES distribution      
 Gene set null distribution of ES for **KEGG\_GLYCOLYSIS\_GLUCONEOGENESIS**
